# The Shared Bicycle and Its Network—Internet of Shared Bicycle (IoSB): A Review and Survey

**DOI:** 10.3390/s18082581

**Published:** 2018-08-07

**Authors:** Shu Shen, Zhao-Qing Wei, Li-Juan Sun, Yang-Qing Su, Ru-Chuan Wang, Han-Ming Jiang

**Affiliations:** 1 School of Computer Science & Technology, Nanjing University of Posts and Telecommunications, Nanjing 210003, China; shens@njupt.edu.cn (S.S.); 1017041027@njupt.edu.cn (Z.-Q.W.); wangrc@njupt.edu.cn (R.-C.W.); 2 Jiangsu High Technology Research Key Laboratory for Wireless Sensor Networks, Nanjing 210003, China; 3 School of Internet of things, Nanjing University of Posts and Telecommunications, Nanjing 210003, China; 1017071608@njupt.edu.cn (Y.-Q.S.); 1217073911@njupt.edu.cn (H.-M.J.)

**Keywords:** shared bicycle, Internet of Shared Bicycle (IoSB), smart city, Internet of Things (IoT), Intelligent Transport Systems (ITS), Wireless Sensor Network (WSN)

## Abstract

With the expansion of Intelligent Transport Systems (ITS) in smart cities, the shared bicycle has developed quickly as a new green public transportation mode, and is changing the travel habits of citizens heavily across the world, especially in China. The purpose of the current paper is to provide an inclusive review and survey on shared bicycle besides its benefits, history, brands and comparisons. In addition, it proposes the concept of the Internet of Shared Bicycle (IoSB) for the first time, as far as we know, to find a feasible solution for those technical problems of the shared bicycle. The possible architecture of IoSB in our opinion is presented, as well as most of key IoT technologies, and their capabilities to merge into and apply to the different parts of IoSB are introduced. Meanwhile, some challenges and barriers to IoSB’s implementation are expressed thoroughly too. As far as the advice for overcoming those barriers be concerned, the IoSB’s potential aspects and applications in smart city with respect to technology development in the future provide another valuable further discussion in this paper.

## 1. Introduction

### 1.1. Concepts

**Figure 1:**
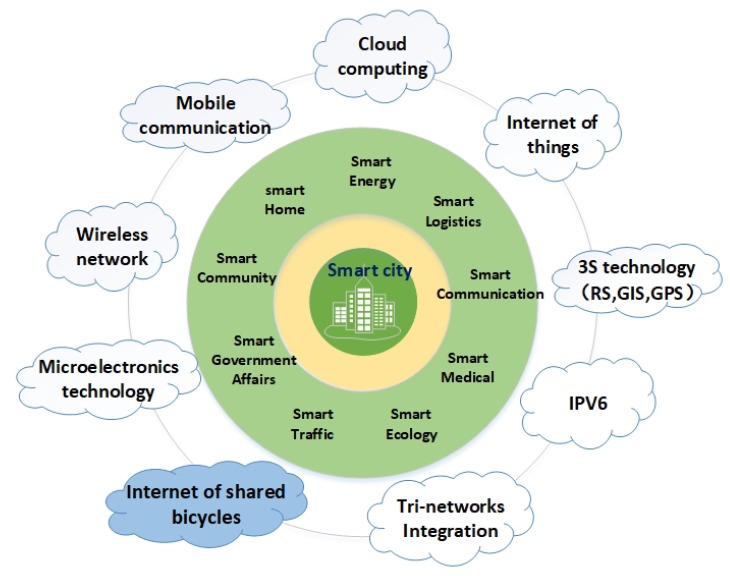
Smart city’s structure.

Because of the rapid growth of the urban population, some severe problems such as environment pollution, traffic jams, and energy consumption, have gained increasing public focus, and become the worldwide issues which almost all cities in any country have to face. In 1987, the World Health Organization (WHO) first proposed the original idea of smart city in a so-called Healthy Cities programme, which is a long-term international development to remind the government decision makers putting the health of the citizens at the forefront [1,2]. Meanwhile, especially in recent years, the development of modern information technologies is enabling cities to be more intelligent and efficient. Through many efforts in the last decades, smart city has a much broader definition, which includes almost every aspect in human beings’ life, e.g., smart medical, smart energy, smart traffic, and smart home, as shown in Figure 1.

As one of the key information technologies, the Internet of Things (IoT), which involves Radio Frequency Identification (RFID), Near Field Communication (NFC), Wireless Sensor Network (WSN), and other related technologies, is playing a more and more important role in the construction of the smart city [3,4]. The core of the concept of the smart city is helping those decision makers to make intelligent and effective city-related decisions through offering them the adequate information at appropriate time and accurate place with the help of all sorts of intelligent devices [5]. As a typical example, an Intelligent Transportation System (ITS) is able to improve the transportation mobility and safety as well as to reduce environmental impact effectively [6]. From the second half of 2016 in China, the shared bicycle, one type of ITS, has developed quickly and is changing citizens’ travel habit a lot. The shared bicycle offers bicycle sharing service based on time-sharing rental model by government and enterprises, which offers citizens many public bicycles at such public sites as subway and bus stations, university campuses, commercial and residential areas. To the best of our knowledge, there are three obvious merits of shared bicycle, as followings:

1. It is an absolute green travel, which could reduce the influence of environment, control the air pollution, and save energy effectively. Without fossil fuel consumption, there is no exhaust emissions which is harmful to the environment. Considering the fact that cycling is also a way of physical exercise, the shared bicycle is beneficial to both environment and users.
2. It provides a perfect solution to the last mile travel. Actually most citizens do not prefer cycling in long-distance travel today. However, taking into account the fact that the range between the subway or bus station and commercial or residential areas is not so long in most cases, shared bicycle is really most economical and suitable in short-distance travel for citizens.
3. As a typical example of shared economy, it is a public resource sharing service, which is able to improve the utilization rate of bicycles. Nowadays, more and more citizens prefer shared bicycle, which is replacing the traditional private bicycle gradually. It will be helpful to ease the traffic pressure as a result.

To unleash all these advantages of shared bicycle mentioned above better, the Internet of Shared Bicycle (IoSB), which is proposed in this paper for the first time as far as we know, is important and valuable to study, in our opinion. The purpose of IoSB is constructing such a framework, which could collect massive data from the thousands of shared bicycles on one hand, and analyse these big data to make long-term development planning for the city on the other hand. IoSB focus on those technologies which are related to wireless communication, positioning, signal sensing, power supply, data analyse, and so on. On this basis, in this paper, a review and survey of shared bicycle and IoSB from related studies and reports is conducted.

### 1.2. Motivations

In the concept of smart city, almost every smart device with good ability of perception is able to interconnect surroundings with the web of internet. As another example of ITS, Internet of Vehicles (IoV), also called Vehicle Ad-hoc Network (VANET) provides a lot of inspirations to IoSB in this paper.

**Figure 2:**
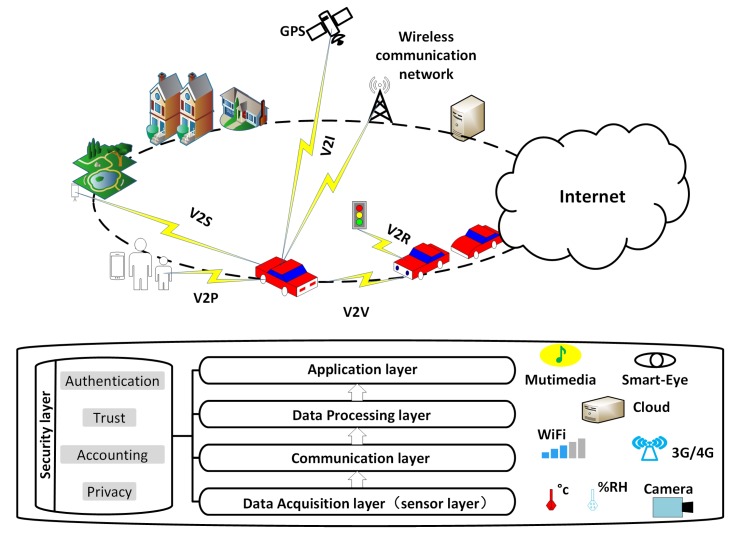
Diagram of IoV.

The communication architecture of IoV includes vehicles, Road Side Units (RSU), and other communication device, as shown in Figure 2. Consequently, the heterogeneous network architecture of IoV has been classified into different modes of vehicular communications, which involve Vehicle to Vehicle (V2V), Vehicle to Pedestrian (V2P), Vehicle to Infrastructure (V2I), Vehicle to Sensor (V2S), and Vehicle to Roadside (V2R) [7,8]. The architecture of IoV is commonly defined as five layers, which are data acquisition layer, communication layer, data processing layer, application and security layer [7,8,9]. As a result of integration of IoT technologies (e.g., NFC, LWPAN, ZigBee, WIFI, Bluetooth), IoV is evolving into a heterogeneous network framework which is playing a more and more significant role in ITS [5]. In future, it will be able to provide a reliable communication platform for various mobile Internet and a pledge on safe driving [10].

**Figure 3:**
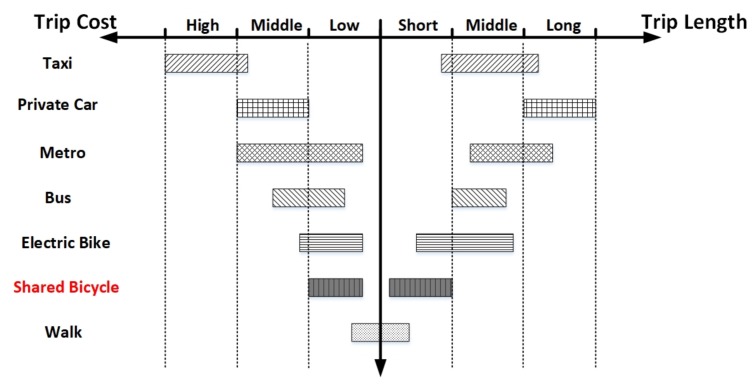
Comparison among different transport methods in urban area [11].

However, the increasing number of cars has brought great pressure to city transportation and eventually resulted in traffic jams. It seems that the shared bicycle is a better solution because of its good flexibility. The trip lengths and costs among different transport methods in urban area are compared in Figure 3. Those public and private transportation systems based on vehicles (e.g., bus, metro, private car, and taxi) are more suitable for mid-distance and long-distance travel. On the other hand, walking and bicycling are more popular in short-distance travel. Compared with walking, it is obvious that bicycling could save more time and physical strength. As a result, when it comes to trip length, cost and trip speed, the shared bicycle performs best in short-distance travel.

Compared with other transport methods except walking, bicycling is more environmentally friendly and low-carbon. The bicycling and walking emit the least amount of carbon under the same parameters, as shown in Table 1. To promote using bicycling and other clean transport methods, the European Commission Decision C 6776 founded the European Transport Specific Programme [12,13].

Last but not least, as a new type of sharing economy like Uber, the shared bicycle allows people to rent a bicycle at a low price for a short period instead of purchasing them. More importantly, the shared bicycle is also a sustainable transport to increase the utilization efficiency of public traffic resources, which is one of the goals in ITS [6]. Whether it is for consumers, operators, governments, and the environment, the implementation of shared bicycle would result in a win-win situation through reusing and recycling of resources [16].

In summary, those three advantages of shared bicycle mentioned above are in compliance with requirements of ITS perfectly [6]. Furthermore, a large scale heterogeneous network can be established with the help of IoT technology to conducted traffic monitoring, air monitoring and other applications in smart city [4].

## 2. Background

### 2.1. History

The shared bicycle, also called bicycle-sharing in some references [11,17,18,19], is about 55 years old and has four generations as far as we know. Especially in recent years, the shared bicycle are flourishing in smart cities, and most of them are successful. Table 2 shows the progress of shared bicycle.

The first generation shared bicycle was firstly introduced in 1965, which provided free bicycles to borrow and return from any location [11]. However, since the fact that the shared bicycle were often stolen or damaged by users, it seemed the safety became a big problem to shared bicycle, which would prevent the further development and raise the maintenance cost obviously [18].

In 1995, the second generation shared bicycle was born in Copenhagen (called Bycyklen or City Bikes [11]) with many improvements over the previous generation. The bicycle consisted of solid rubber tires and wheels, and could be borrowed and returned from any self-service bicycle station throughout the city with coin or smart cards access [17]. The bicycles of this generation were equipped with a docking station, which is the prototype of the modern public bicycles.

Shared bicycle of the third generation appeared in 2006. In this generation, Global Positioning System (GPS) was utilized to provide the reasonable locations, which was helpful for better tracking of bikes [17]. Another innovation was recording the riding time and provide it to users at specific station with the help of the new network and information service [11,24]. In addition, combined with Geographic Information System (GIS) [21], the distribution of bicycle can be analyzed and the plan of bicycle scheduling could be simpler. With the possibility of networking and information service, this generation alleviated or solved some problems. For example, it was possible to get users’ information before lending the bicycles, and some related companies were able to check the user’s historical transaction. In 2009, some operators used a networked bicycle station and RFID technology to monitor the geographical location of shared bicycles (New York City Department of City Planning, 2009) [11].

With the introducing of many new technologies, the shared bicycle of the fourth generation, which is considered to be one type of intelligent hardware, developed so quickly in recent years. Especially with the emergence of sharing economy like Uber in 2010 [31], the public bicycle, which had not been developed smoothly in many cities these years, was becoming popular in a new mode called bicycle-sharing. In April 2016, Mobike launched its smart bike sharing service in Shanghai, which is an attempt to extend the mobile payments to shared bicycle [32]. It is easy to unlock the shared bicycle using QR-code for citizens. In July 2017, another bicycle-sharing brand OFO, along with China telecom and Huawei, launched their smart lock equipped with an Narrow Band Internet of Things (NB-IoT) module [33]. The introduction of NB-IoT technology [34] is expected to greatly reduce the energy consumption of bicycle-sharing system. In addition, there are also some related works on the analysis of big data generated by bicycle cycling. For example, the authors in [35] used crowdsourced data from bicycle-sharing system to analyze the relationship between different cycling purposes and air pollution exposure. The results shows that cyclists riding for recreation and other purposes tend to be exposed to lower levels of air pollution than cyclists riding for commuting, which could be an inspiration for policymaker to consider how to improve cycling infrastructure and road safety. Except for Strava Metro data (the data sources from [35]), OFO and Mobike companies in China have also set up their own big data platforms which data sources are based on their own shared bicycles [36,37].

### 2.2. Brands and Market in China

It is nine years since the shared bicycle went into China. Its development could be divided into two phases in general. The first phase, belonging to the third generation presented before, is mainly the development of shared bicycle with dock since 2008 to 2014 [38]. A typical example of this generation is the public bicycle operated by the government. Since the public bicycle is based on dock, many problems arose and were summarized in [39], such as fragile bicycles, irrational distribution of dock stations, inadequate capacity of dock stations and so on. The second phase, which is the fourth generation we mentioned above, is developing without dock since 2015. There are so many brands of dockless shared bicycle in China’s market today, such as OFO, Mobike, Bluegogo, and so on. The situation of public bicycle in Hangzhou city (HZBike), OFO, and Mobike are summarized in Table 3.

#### 2.2.1. HZBike

To deal with the severe air pollution problem, Chinese government has introduced into the public bicycle programs in several large cities, for example, Beijing, Wuhan and Hangzhou. Among these programs, Hangzhou public bicycle (HZBike) is most successful, which won the 2017 Ashden Awards because of creating a sustainable transport system [40]. HZBike is a bicycle-sharing program by Hangzhou government, which uses smart-card and RFID for check-in and check-out. HZBike is still improving their products to meet the users’ requirements. There are some highlights of HZBike model, which are summarized as followings in [40].

- Bicycle docks distribute scientifically.
- The smart card can be used across all public transport systems, including taxis and public bicycle.
- There is an integrated and efficient transfer system in Hangzhou city.
- The cost is very low in using.
- It is a government-led business model.
- A complete real-time monitoring and arrangement system is used.

#### 2.2.2. OFO

Without a dock, it is possible to park OFO bicycles anywhere. Until now, there are three generations about OFO bicycles according to their different locks. The OFO bicycle of first generation with a 4-digit mechanical lock, was born in some universities campus in China in 2015. Considering the low security about mechanical lock, at the end of 2016, OFO launched the bicycle of second generation with a smart lock, which was equipped with GPS tracking device and was charged by solar panel. To improve the convenience, Quick Response (QR) code could be scanned by user’s mobile phone to unlock the bicycle. In August 2017, the union of OFO, China Telecom and Huawei, proposed the third-generation bicycle with NB-IoT-based smart lock to realize the lower energy consumption and stabler network connection.

#### 2.2.3. Mobike

As another leading brand of shared bicycle in China, Mobike, which has two kinds of bicycle, Classic Mobike and Mobike Lite, is very popular among youth. The Mobike of the first generation was launched since April 2016 in Shanghai. At the beginning, it already had many fashionable design such as aluminum alloy body, solid tires and rear-wheel-based power generation. To solve such problems as overweight body and high production cost, the second generation applied a more durable aluminum kickstand. Another remarkable improvement was introduction of the new hydraulic adjustable seat.

### 2.3. Comparisons

Inspired by some investigation work in [11], we presented the performance of current brands of shared bicycle in five aspects to have an intuitive comparison, which are convenience, user fees, cycling comfort, stability and security that can greatly influence users’ willingness to ride. In this context, these five aspects are compared among HZBike, OFO, and Mobike. Here we use a score between 1 and 3 to evaluate the performance of every brand in every aspect, which means that the higher score indicates the better performance.

#### 2.3.1. Convenience

**Details:** The convenience involves all aspects of bicycle rental, including account application, bicycle searching, bicycle returning, fees payment, etc.

**Ranking:** Mobike (3) = OFO (3) > HZBike (1)

**Reasons:** First of all, the handle procedures of the cycling card for HZBike are complicated and only available in some manual service points in Hangzhou. In contrast, the qualification of OFO and Mobike can be achieved through several simple steps in mobile app. Compared with the fixed parking pile of public bicycles, the dockless mode of OFO and Mobike seems to be more casual and convenient for users to return the bicycle. Additionally, users who rent a Mobike or OFO can pay their fees using e-payment, such as Alipay or WeChat Pay that is much more convenient than a traditional bus pass or cash. Similarly important, OFO and Mobike are available in most cities in China which means users who has an account can rent an OFO or Mobike when they go to other cities. However, for a kind of public transport dominated by the government, HZBike cannot be so convenient like OFO and Mobike for some reasons.

#### 2.3.2. User Fees

**Details:** The price includes deposit and rent.

**Ranking:** HZBike (3) > OFO (2) > Mobike (1)

**Reasons:** The price of the three brands are listed in Table 4. In deposit, Mobike needs 299 CNY, while 199 CNY for HZBike and OFO. Citizens can take a free ride on HZBike in one hour. Afterwards, the rent of HZBike is 1 CNY per hour. Generally speaking, the rent of OFO and Mobike is 1 CNY. Because of a promotion for campus by OFO, teachers and students could enjoy a lower rent, 0.5 CNY per hour. By the way, OFO and Mobike often launch some activities to offer the users many rent discounts.

#### 2.3.3. Cycling Comfort

**Details:** As an intuitive feeling of users, cycling comfort depends on the design and materials of the bicycle and can greatly influence users’ choice of bicycle brand and. The ranking below is based on three aspects which are weight, tire and seat (see Table 5).

**Ranking:** HZBike (2) = OFO (2) > Mobike (1)

**Reasons:** First of all, the weight of a bicycle should be taken into account. Through field measurements or relevant data on the Internet, we get the weight of 3 types of bicycles in Table 5. Due to the difference in structure and materials, Mobike weighs more than 20 kg, while the other two weigh less than 20 kg. Secondly, compared with the HZBike equipped with traditional pneumatic tire, the OFO and Mobike, which use solid tire, have poor anti-vibration capability and are harder while cycling. Finally, because of different body size of users, an adjustable seat will enable them to maintain a comfortable posture while cycling. At this point, the two new shared bicycle (OFO and Mobike) are doing better than HZBike.

#### 2.3.4. Stability

**Details:** The stability is mainly related with the quality of bicycle and the robustness of the renting system, which also affects the maintenance strongly.

**Ranking:** HZBike (3) > Mobike (2) > OFO (1)

**Reasons:** First of all, the dockless model adds great difficulty to manual maintenance, even if they are equipped with GPS positioning equipment. By comparison, the traditional public bike is much better, and the excellent operation of the HZBike has made it one of the most popular public bike systems in the world [40]. In terms of body materials, limited by manufacturing costs (see Table 6), OFO and HZBike are made of common iron, which is more vulnerable to corrosion and the performance of components in bicycle will decrease, even broken.

#### 2.3.5. Security

**Details:** The security refers to not only the personal safety of cyclists, but also the information security of their accounts.

**Ranking:** HZBike (3) > Mobike (2) > OFO (1)

**Reasons:** Most cycling accidents are related to the braking system of bicycle. We have investigated the brake types of 3 brands, as shown in Table 7. Among them, the best braking performance is the Mobike that equipped with disc brake, followed by OFO, and finally HZBike. Furthermore, to avoid children cycling accident, three brands all have a real name registration system. In terms of payment of shared bicycle, the emerging QR-code still has security risks compared with RFID. In China, there is a kind of fraud that a QR code with Trojan virus is stuck on the bike to cover up the real QR code and make the key information of the users’phone stolen, which is more likely to happen on the shared bicycles like OFO and Mobike. Furthermore, HZBike is a part of Hangzhou city traffic and is directly regulated by the HangZhou municipal government, which makes user’s deposit and personal information more secure.

**Figure 4:**
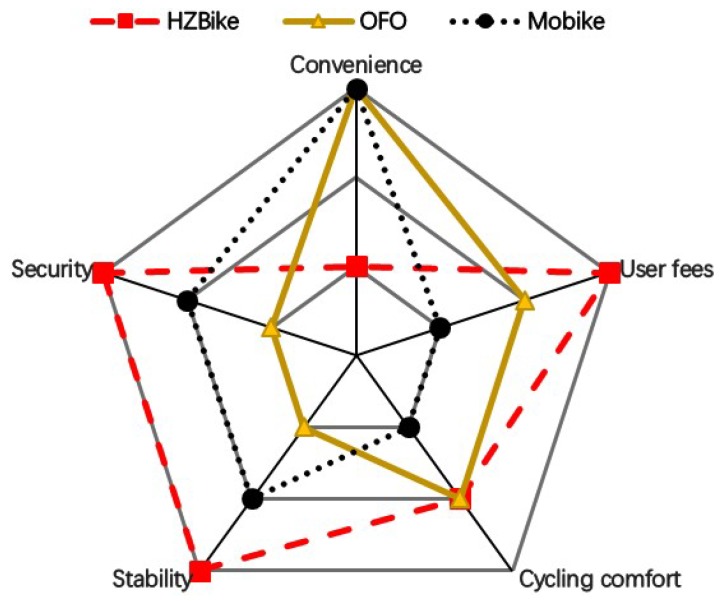
Comparison among HZBike, Mobike, OFO.

The comparison results are indicated in Figure 4 below. As can be seen from Figure 4, traditional public bicycles still have advantages in security, stability and user fees. Whereas, in terms of convenience, the emerging sharing bike has an absolute advantage due to the dockless model. By contrast, OFO focuses on cycling comfort and user fees, while Mobike focuses on stability and security. However, the above comparison is only a temporary situation. With the fierce competition in the market of shared bicycle, the three brands are making up for their shortcomings and improving constantly. For example, HZBike will be equipped with a QR-code, solid tire and GPS positioning [44]. OFO and Mobike have also introduced safer NFC payment technology and lighter bodies [45,46].

## 3. Key Technologies of IoSB

### 3.1. Architecture of IoSB

Actually, bicycle system based on the IoT technology has been proposed in some work before. For instance, a Smart E-bike Monitoring System (SEMS) was proposed in [47], which is loaded with batteries, motors, GPS devices and other sensors and supports a real-time usage and environment perception. Besides, an IoT solution designed for public bicycle system to collect the energy from the bicycle Dynamo Hub itself and localization scheme through 3.5 G mobile network is proposed in [48].

**Figure 5:**
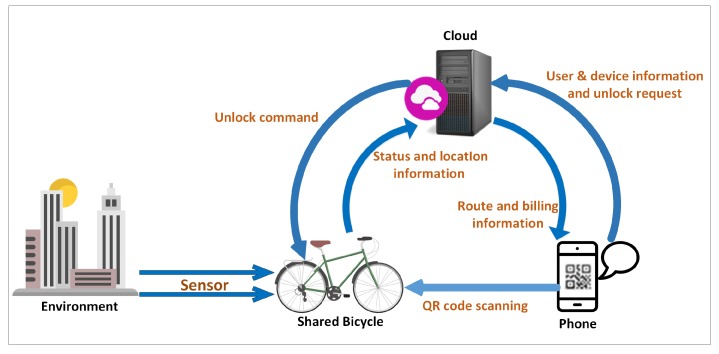
Purpose of IoSB.

The present shared bicycle system in China has been taking shape gradually since the second half of 2016. As is shown in Figure 5, there are four interconnected objects in IoSB architecture in our opinion, which are environment, bicycle, phone and cloud platform. Except for the information generated by users’ cycling, some ambient information are valuable and can be sensed and uploaded by shared bicycle, and finally be processed in cloud platform, which is a typical and technically accessible IoT application scenario.

**Figure 6:**
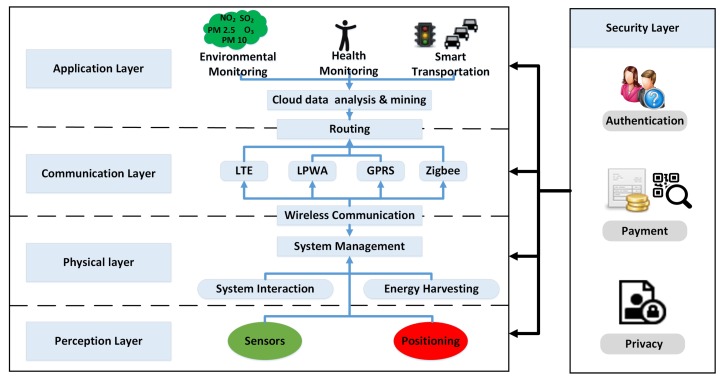
Proposed architecture of IoSB.

The architecture of IoSB should be able to realize some real-time interactions with users to complete such operations as bicycle unlocking, positioning and data uploading, which would consume a lot of electricity. Therefore, a power-saving and service oriented architecture should be given. Therefore, inspired by the work in [47,48,49] and the development of shared bicycle in China recently, a five-layered model architecture of IoSB, which includes perception, physical, communication, application and security layers, is proposed in Figure 6 for the first time as far as we know.

**Perception layer:** The first layer is consisted of some sensors, positioning module and some other data-acquisition modules. The primary responsibility of this layer is gathering information regarding environment, traveling routes and cyclists’ health. The vast information includes air quality, speed, position, health information of cyclist, weather conditions, and multimedia information. Meanwhile, collection and differentiation of captured information should be implemented in an efficient manner in terms of cost and energy.

**Physical layer:** The second layer is mainly related with those support work for the system, which includes energy harvesting, system interaction and system management. Because of the support for system’s stability and interactivity, this layer has great value for the whole IoSB. Moreover, due to the close relevance between interaction process and verification of the users’ identity, the design of this layer is critical to ensure security of shared bicycle that mentioned before.

**Communication layer:** The third layer is mainly responsible for the uploading of information with low delay and high QoS level. In this layer, information is transmitted between point and point according to the routing protocols until uploaded to the server. Relevant transmission technologies in this layer include LTE, Zigbee, LPWA, GPRS and so on. Considering the limited energy of nodes in IoSB, a less power-consuming information transmission technology should be selected.

**Application layer:** The primary missions of this layer are information processing and command issuing. Besides completing such key operations as bicycle unlocking, user authentication, cycling data management, account deduction, the cloud sever also need to process the huge amounts of data collected by bicycle node. Accordingly, some application fields like environmental monitoring, health monitoring and smart transportation will benefit from the data analysis results.

**Security layer:** This is a transversal layer that has direct relation with the other layers. It is responsible of some security functions, such as account authentication, fee settlement, user information update and maintenance, etc. In addition to enabling the cloud server to resist some network security attacks, the design of its mobile app should also provide users with a safe payment environment and stable system services.

**Figure 7:**
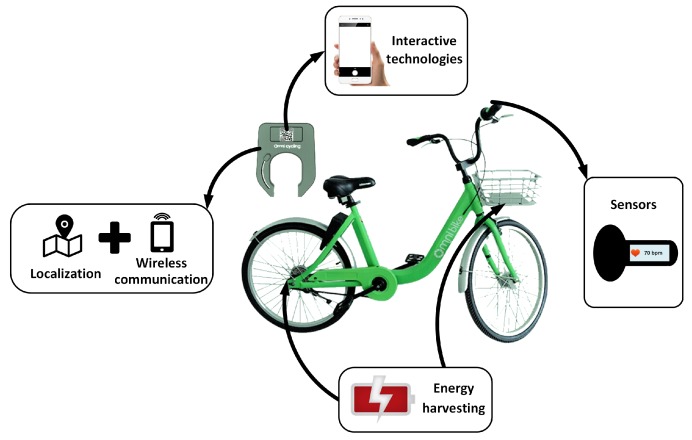
Key technologies in IoSB.

As a complex heterogeneous network, IoSB contains many key technologies, such as sensor technology, positioning technology, interactive technology, energy-harvesting technology, communication technology and data mining technology (see Figure 7), which will be discussed in followings.

### 3.2. Sensors

A sensor is a device that converts information into electrical signals. In IoSB, the limited energy and space of bicycle limit the number of sensors that can be assembled. As can be seen from a survey in [50], people are paying more attention to the sensors’ dependability, size, volume, mass, installed longevity and some other capabilities, which would be the development trend of sensor in the future.

Commonly used sensors are environmental sensors [51], body sensors [52] and multimedia sensors [53]. Environmental sensors, which are generally small enough to be embedded in a bicycle, can extract temperature, humidity, the concentration of some hazard gases or particulate matter (e.g., SO${}_{2}$, CO${}_{2}$, PM2.5 and PM10) and some other parameters from environment [54,55]. For example, our work in [56] designed an air quality monitoring system based on WSN.

Body sensors are designed to monitor human health parameters including body or skin temperature, oxygen saturation, ECG, respiration rate and so on [52]. With the development of microelectronic technology and the rise of Body Area Network (BAN) [57,58], Body sensors become smaller and more energy efficient, which is ideal for small embedded systems like IoSB. For instance, some spinning bikes or smart watches today are equipped with an optics-based heart rate measurement tools to monitor users’ health in real time. In addition, multimedia sensors can also be used in embedded devices. However, considering its higher power consumption and bigger data packets, it is hard to apply multimedia sensors to resource-limited IoSB.

### 3.3. Localization

In fact, IoSB is an application of Mobile Wireless Sensor Networks (MWSNs) [59]. Different from the localization of static nodes in WSN, the nodes in MWSNs will move irregularly, which makes it harder to get location. Meanwhile, multipath and Non-Line-of-Sight (NLoS) propagation are always underlying causes to affect the performance of location [60]. The commonly used mobile localization methods include Global Positioning System (GPS), Cellular Positioning System (CPS) and Wireless Local Area Network (WLAN) positioning system [61].

As one of the earliest application of positioning technology, GPS positioning performs quite well. At present, most of shared bicycles are equipped with a GPS module for positioning. However, with GPS alone, positioning accuracy is not enough. To further improve the bit rate of the navigation data encoded in the signals transmitted by the satellites (one of the main disadvantages of GPS), Assisted GPS (A-GPS) is designed to reduce the startup time by fetching the navigation data over the Internet, commonly by connecting via a cellular network [62]. By combining with the cellular positioning system (CPS), this system (A-GPS) can locate the caller to within 50 m for 67% of calls and to 150 m for 95% of calls [63]. CPS location accuracy depends solely on the cell size, but this can be enhanced with support of other techniques, such as sector division by directional base station antennas and Received Signal Strength (RSS). With more dense base stations distributed in the upcoming 5th generation wireless systems, the combination of GPS and CPS will greatly improve the accuracy of mobile positioning, which is expected to be the trend towards shared bicycle positioning.

In the complex urban environment, indoor positioning technology has always been a pain point for the existing shared bicycle. Currently WLAN positioning system is the dominant indoor positioning due to the IEEE 802.11 standard’s wide application and can make up for the defects of positioning technology in existing IoSB. Historically, from the position computation perspective of radio-based signaling systems, the known approaches for WLAN positioning can be divided into three main types [64]: (1) Angle of Arrival (AoA) and related Direction of Arrival (DoA) methods; (2) Time of Arrival (ToA) and related Time Difference of Arrival (TDoA) techniques; (3) RSS exploitation methods (fingerprinting). In terms of positioning performance, the accuracy of typical WLAN positioning systems using RSS is approximately 3 to 30 m, with an update rate in the range of a few seconds [65]. However, it is still a little difficult to apply WLAN positioning systems to shared bicycle, which involves power consumption, positioning chip design technology and so on.

### 3.4. Interactive Technologies

The interaction between shared bicycles and users involves not only the user’s account security, but also the interests of the service provider. Therefore, some specialized interactive technologies should be applied in IoSB, which can make the process as easy and efficient as possible while ensuring security. Some technologies used for mobile payment may work, such as QR code or barcode in Alipay [66], Near Field Communication (NFC) [67], and Bluetooth. On the other hand, acoustic communication (just like sound pay in Alipay) could be chosen to be a bicycle unlock way too [68].

QR code is a type of matrix bar code or two-dimensional code that can store data information and designed to be read by smart phones [69]. In IoSB, people can use a smart phone to scan the QR code on the shared bicycle and then transmit a unlock request to cloud sever. However, QR code also have some security flaws, as mentioned in the previous chapter on the discussion of bicycle security. Unlike QR code, NFC technology can provide a secure form of authentication that is being used in smart cards and mobile authentication [67,70]. For example, in September 2017, OFO launched a bicycle with NFC module, and compared with the QR code unlock scheme, NFC has advantages in user experience, data transmission and security. Besides, Bluetooth also known as the IEEE 802.15.1 [71], can be used for establishing Personal Area Networks (PAN) communication and unlocking the shared bicycle securely in IoSB. However, for the shared bicycle unlocking, the process of Bluetooth connecting is cumbersome and waste a lot of time as a result.

### 3.5. Energy Harvesting

In IoSB, bicycle nodes are battery-powered and often dispersed in the city that is hard to access for a battery replacement. For overcoming the problem of partial node death due to exhaustion of energy, energy-harvesting technology is particularly important. A comprehensive introduction of ambient sources for energy-harvesting has been given in [72].

Solar energy is richly available in nature which is green and free of cost. A solar cell can convert the sunlight energy into electrical energy and stored in battery in form of chemical energy for the further use [73]. In [74], a solar energy-powered bicycle system was designed for the monitoring of immediate solar degree of illumination and ambient temperature. Existing shared bicycle like Mobike and Hellobike, are all using this kind of energy-harvesting technology. However, it has the disadvantage of being able to generate energy only when there is sufficient sunlight or artificial light.

Vibrational, kinetic and mechanical energy generated by movements of objects can also be harvested. One method of harvesting mechanical energy (e.g., mechanical energy of cycling) is through the use of a dynamo installed in bicycle hub, which has a very high energy conversion rate but poor user cycling experience. In the classic Mobike, the installation of dynamo results in heavy bicycle body and hard riding.

Ambient RF energy has a relatively low energy density compared to other energy sources [75]. However, owing to the rapid development of cellular networks, the ambient RF power density in city is getting higher and higher. The RF energy-harvesting technologies is especially useful in charging a battery or powering up electronics by wireless in scenarios of the deployed wireless networks, just like IoSB. However, some of its problems, such as low energy conversion rate and low coverage, remain to be solved [76].

### 3.6. Wireless Communication

Reliable transmission of information is the guarantee of data application, especially for real-time application just like shared bicycle. The quality of communication is affected by communication standard, network topology, base station capacity, etc. The following part will introduce some relevant communication technologies.

Long Term Evolution (LTE) and 3G are standards for wireless communication on all user mobile phones, and they are also standards for a high speed on data terminals [77]. Taking into account of the development of wireless communication infrastructures all over the world, especially in developing countries, these technologies are available everywhere, suitable for broadband connectivity and long range applications, such as IoSB. However, for now, 3 G/4 G communication modules have higher cost and power consumption, which is overkill for IoSB in terms of transmission performance.

Low Power Wide Area (LPWA) technology is designed for the transmission distance more than 3 km in the complex urban environments and 15 km in open area with strong penetrability. Compared with 3 G/4 G communication, LPWA is more suitable to be applied in scenarios such as tracking, smart parking, smart agriculture, and shared bicycle. NB-IoT and eMTC are wide-area IoT technologies that can be integrated with existing cellular networks and features of low cost, high reliability, and high security [78]. NB-IoT, which has been used in the latest generation of OFO bicycle, has four advantages: (1) Super coverage; (2) Low power; (3) Low cost; (4) Massive connection [34]. With the maturity of network standards and related hardware, NB-IoT has been used in monitoring of water level [79], smart hospitals [80], car parking system [81], etc.

## 4. Challenges

**Figure 8:**
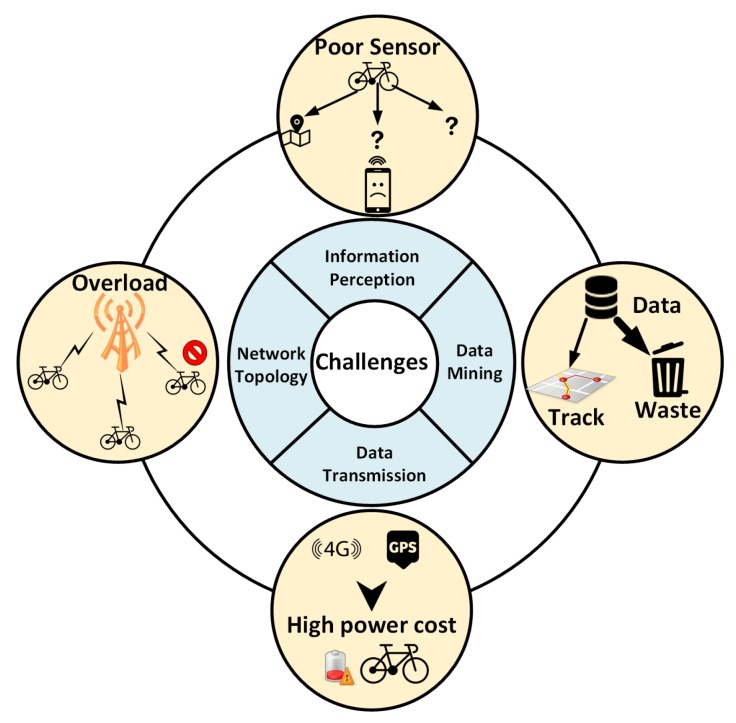
Challenges of IoSB.

The development of shared bicycle is so fast, but incomplete and problematic. In terms of the perception and processing of data, some obvious shortcomings existed in current IoSB. This section will present some current challenges about techniques in IoSB, which is shown in Figure 8.

### 4.1. Single Type of Information Sensed

In IoSB, thousands of shared bicycle nodes are distributed in every corner of the city and are closely related to citizens’ daily travel, which is suitable for urban IoT. However, now, what can be perceived in IoSB is just the location of bicyclists and some information obtained through further analysis like researches in [82,83], which seems to be too scant. As a result, a lot of other valuable data that can be of great value to smart city such as environmental information [2,84,85], health information of drivers [86,87], and multimedia information [53] (including photo, sound and video) is unavailable according to current technical scheme. Moreover, the nodes in the current IoSB cannot withstand the power supply of multi-sensor system. Therefore, the key issue is how to perceive the location, environment, physical signs and other kinds of information on a shared bicycle node with limited resources and energy.

### 4.2. Simple Clustering and Routing Methods

In the current shared bicycle system, the network topology is a simple star structure and data is transmitted directly to the cloud server in a mode of point-to-point and single-hop. As a result, the range of network coverage is limited and a huge load will be brought for the base station, which seems to be inflexible and has no benefit for a robustness WSN [88]. In smart city, some topology control algorithms listed in [89] like CDS, EECDS and so on, can evidently improve the coverage and energy efficiency of the whole network. Then, considering the characteristic of the network architecture of IoSB, a corresponding efficient topology control algorithm should be proposed to realize the collaborative perception and calculation among mobile nodes.

### 4.3. Non-Collaborative Mode between Communication and Localization

As two key parts of IoSB, communication and localization should be in a close correlation with each other. Some methods to improve positioning through communication between nodes are summarized in [90] (also known as cooperative localization), which turns out to increase the localization accuracy and decrease the time delay [91]. However, most of the current shared bicycles in China like OFO and Mobike employ an absolutely separated scheme, which applies 3 G/4 G cellular network in communication and GPS in positioning, and there seems no correlation between them. As a result, some problems may be caused, such as high power consumption, poor location accuracy in some places without Line-of-Sight (LoS), a high load on base station, etc. Consequently, an improved organization of communication, positioning, and even wireless power charge would attract more attentions to achieve a novel collaborative mode.

### 4.4. Insufficient Data Mining

Big data applications refer to not only the collection of massive data, but also the analysis and mining of data. Massive data sensed by shared bicycles can be applied in many aspects, such as urban planning, traffic management, environmental monitoring, etc. For instance, a research of bike lanes planning based on Mobike’s trajectories was carried on [82] in 2016, which has sufficiently considered three constraints and requirements from urban planners’ perspective. In addition, in earlier times, some analytical work on bicycle station usage from Barcelona’s shared bicycling system was presented in [92]. However, due to the advance of shared bicycle nowadays, excavation of the massive data collected by shared bicycles is far from adequate. It would be so valuable to fuse more kinds of data and apply it in more aspects that can increase the well-being of residents. Therefore, much more further studies on application scenarios of shared bicycle in smart city are required, as well as its data analysis method.

## 5. Future Aspects of IoSB

### 5.1. Information Perception

For better sensing and predicting the pulse of the city, a solution to the first problem in the previous chapter should be given. Some schemes addressed the energy efficiency through the minimization of the output average bit-rate. For example, a highly-digital frequency-domain implementation of a Level-Crossing-sampling (LC) Analog-to-Digital Converter (ADC) was described in [93], which aims at increasing amenability to ultra-scaled CMOS technologies. Some new ideas are also expected to solve the energy consumption problem of multi-sensor nodes.

In [94], D.L. Donoho firstly proposed an influential signal processing method-Compressed Sensing (CS), which can greatly reduce the complexity of signal acquisition end. Different from traditional ADC, Analog-to-Information Converter (AIC) is the hardware implementation method that applies CS theory to practical application, and can promise that the original signal can be reconstructed with a large probability at the signal receiving end in the case of low sampling rate at the signal acquisition end. In [95,96], RICE University in the U.S. produced a single-pixel camera based on the principle of AIC, using a mirror array with an adjustable angle of reflection to complete the matrix operation of the optical image, which verifies the feasibility of AIC.

**Figure 9:**
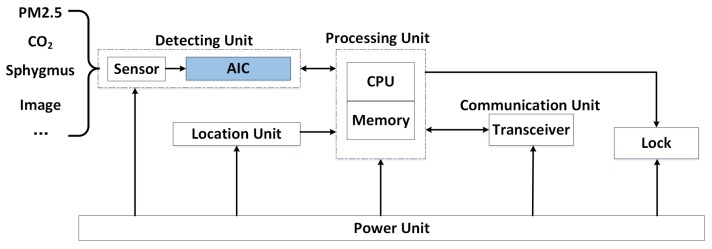
An AIC-based system structure of the sensing node in IoSB.

Based on the above considerations, we give a system structure of the sensing nodes in future IoSB for the first time, which adopts the AIC compression sampling technique and includes communication unit, processing unit, location unit, detecting unit, lock and power unit in Figure 9.

### 5.2. Network Topology Control

To improve the coverage and energy efficiency of the whole network, dynamic clustering and routing protocols are required. The author in [97] proposed a two phase dynamic method for cluster head selection in WSN. Their technique is suitable for stationary sensors. In contrast, our work focus on IoSB composed of mobile nodes. In [98], Optimal Clustering Algorithm (OCA) is expected to increase network lifespan and dynamic. The authors in [99] design and implement a new dynamic topology for mobile sensor networks, called Dynamic Broadcast Clustering Topology (DBCT). In their approach, the network topology has better feasibility and mobility. Differing from the above DBCT schemes, Ref. [100] exploited a topological control method for Chain-type Wireless Sensor Networks (CWSNs) which can evidently improve the coverage of the whole network. The literature [101] exploited a new robustness enhancing algorithm for scale-free WSNs, which can improve the robustness of the network.

**Figure 10:**
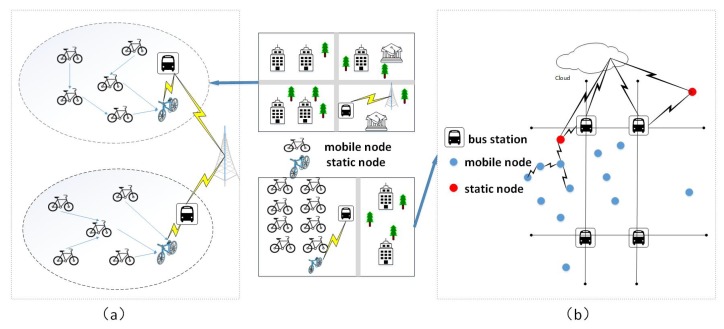
Network deployment and work schematic of IoSB, (**a**) hierarchical clustering heterogeneous network; (**b**) topology structure of hierarchical clustering heterogeneous network .

Different from the design of mobile sensor networks, such as IoV, we consider the IoSB has its own features, like low speed, limited range of movement, etc. Based on the above considerations, we conceive a hierarchical clustering heterogeneous network, which is shown in Figure 10a. Moreover, considering the ever-changing network topology of IoSB owing to the movement of nodes, we put forward a topology structure of hierarchical clustering heterogeneous network in Figure 10b. Three parts are included in this structure which are static sensor, mobile sensor and bus station. In our vision, the bus station is utilized as a fixed node and carries higher precision sensors compared to mobile node, which can be used for a data transmission relay or location beacon.

### 5.3. Data Transmission and Localization

For easier use and maintenance, each node in IoSB should provide accurate location information at any time and place. Therefore, a comprehensive solution for the third problem mentioned in the previous chapter should be proposed. In [102], a device tracking strategy in NB-IoT systems using Observed Time Difference of Arrival (OTDoA) measurements was presented, which can consume less power because of using NB-IoT [103]. In this strategy, the accuracy evaluation of mobile node in EPA and ETU channel models (corresponding to low and high delay spread environments respectively) were given and results showed that the maximum positioning error was about 300 m when moving at 10 km/h (close to cycling speed). More importantly, narrow band technology can bring massive capacity. The authors in [104] combine D2D link with NB-IoT for a routing extension and enable direct communication between nodes, which is expected to guarantee QoS in NB-IoT systems.

**Figure 11:**
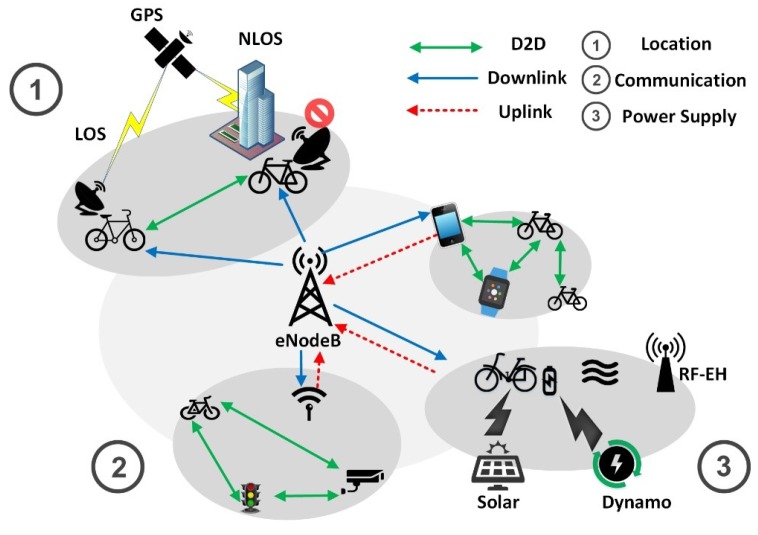
Green IoSB vision in future.

Based on the above discussions, we give the future positioning and communication scenarios in IoSB in Figure 11. There are three parts in Figure 11 which are hybrid location, D2D-based NB-IoT communication and mixed power supply. In the NLoS condition, the bicycle node can be located by other nearby bicycle nodes. In addition, thanks to D2D communication, nodes can upload information to base stations in a multi-hop mode. Furthermore, multi-source power supply shown in Figure 11 can greatly prolong the life of a node. However, further study on device discovery, resource management, and mobility, security and privacy in D2D communication should be done beforehand [105].

### 5.4. Data Mining

A suitable information mining strategy and their application scenarios are critical and should be considered to figure out the last challenge in Section 4. In [106], the authors rationally analyzed the relationship between some environmental factors and the usage of a Bicycle-sharing System (BSS) with a mathematical model, so as to select the appropriate point for a new docking station to increase the usage of BSS. Similarly in some big data analysis of cycling released by OFO and Mobike [107,108,109], the links between some cycling data including cycling duration, cycling moment, geographical location, cycling path, user gender and age, have been analyzed. The results can directly reflect some characteristics about the city’s travel, such as travel peak, pace of city life, user age composition, etc.

**Figure 12:**
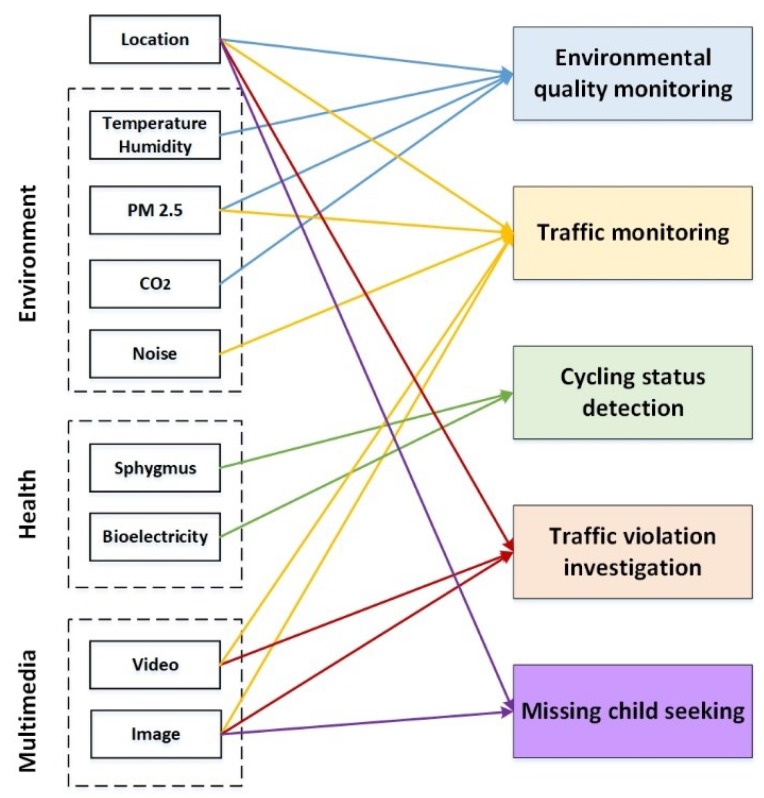
Application scenarios and data correlation in IoSB.

Considering the above cases, correlation analysis of data in IoSB should be done first. Referring to the relevant work in [86,92,110,111,112], we have shown some feasible application scenarios of IoSB and the data associated with them in Figure 12.

Deep learning has been widely used in image recognition, natural language processing and speech recognition and can be a good choice. In [86], a fusion method based on human body sensor networks and vehicular Ad hoc network was designed to identify four dangerous situations in the driving process (including drowsiness, drunkenness, abnormal driving and distracted driving) and send out alerts in time. On the basis of the theory, the corresponding experimental driving was conducted to verify, and the real-time monitoring program based on the Android system was developed to monitor the concentration of attention by Electroencephalogram (EEG). However, due to insufficient study on relevant aspects in shared bicycle, improvements to the classical neural network model should be implemented to adjust IoSB. In [112], a high-precision real-time license plate detection method based on Convolutional Neural Network (CNN) is proposed to achieve higher accuracy and lower costs. As shown in Figure 12, the above CNN-based detection method can also be applied to traffic violation investigation based on the image or video collected in IoSB. In [111], a Multi-layer Perception (MLP) deep learning model for congestion prediction is proposed, and Linear Regression (LR) model is added to predict its duration which data sources are from the Singapore Land Transport Authority, Google and Open Maps weather APIs. Experimental results show that the MLP-LR model performs very well in predicting congestion, with an accuracy of 63%. Analogously, it is feasible to replace the data source of the above method with the data collected by IoSB, and the improved method based on the MLP-LR model mentioned above can be used in IoSB to predict congestion and arrange travel plan.

Further data mining and analysis we are able to do about urban planning, traffic management, and urban security are summarized as follows:

1. Combining location data and air monitoring data, we can geographically divide the city into several regions based on the level of pollution, which will help the environmental protection department to find the source of urban pollution and formulate targeted solutions.
2. From the location data of shared bicycles, we can study the daily routine of citizens, so as to better plan the public transportation schedule, such as the start and end time of the bus and subway, as well as their daily frequency. Moreover, the research on the cycling path of shared bicycle can make the urban infrastructure planning more reasonable and efficient.
3. By collecting electrical signals and age data (obtained from a real-name registered app account) from cyclists, an assessment of their health can be made, so as to analyze the physical condition of users at different ages.
4. Due to the wide range of data sources, the shared bicycle can serve as a remarkable platform for some crowdsourced tasks, such as seeking lost children, arrest of a criminal, inspection of shared bicycle damage status, real-time reporting of traffic conditions and so on. Among them, most of the existing shared bicycles are maintained daily through the information of faulty bicycle reported by users, which is a typical crowdsourcing model. For example, a crowdsourced task can be released to some cyclists who are at a certain geographical location that time to ask them to take a photo that can reflect the congestion on the road at that location. If cyclists can complete the crowdsourced task successfully, several times of right for free cycling or electronic red-envelope can be a reward to motivate cyclists.

## 6. Conclusions

The intelligent transportation is undoubtedly one of the most important parts of smart city. The aim of this paper is to give a comprehensive introduction to shared bicycle and explore development trends of its technologies in the future. In this paper, we firstly introduced the development of shared bicycles around the world, and described some latest changes in China via the comparison among HZBike, Mobike and OFO. Inspired by the IoV, we correspondingly attempted to propose the concept of IoSB and a five-layered model architecture, along with a brief to some relevant technologies. Likewise, some technical challenges arising from implementing the IoSB in China were accordingly illustrated, which involves in the process of information collection, preprocessing, transmission, analysis and mining. Afterwards, some researches on promising solutions to the challenges were also introduced, such as compressed sensing, NB-IoT, and so on. Moreover, we also covered some of the future applications of IoSB. In the data mining part, we first introduced several cases of data analysis on shared bicycle cycling data and then analyzed how to mine cycling data using deep learning method, as well as its difficulties. In addition, we also listed some potential research directions we intend to implement about data analysis using IoSB.

For the future development of IoSB, we believe that the upcoming 5 G technology will bring lower communication time-delay and more accurate positioning. Thus, location-based services such as electronic fence and cycling path record can be better realized. Secondly, there will be more sensors embedded in the shared bicycle with the development of sensor and battery technology, which means more data will be generated about cycling. With some data mining methods, these data can be transformed into more intuitive and understandable forms for decision makers to accept, so as to make the city run more efficiently.

## Figures and Tables

**Table 1:** Carbon emissions of different transport methods [14,15].

Transport Method	Carbon Emission (gCO${}_{2}$/Km/One Person)
Private Car	501
Taxi	167
Metro	60
Bus	20
Shared Bicycle	0
Walk	0

**Table 2:** Progress of shared bicycle.

Gen.	Examples (Name, City or Country, Year)	Features	Defects
	White Bicycle, Amsterdam, 1965 [11];	Low production cost;	Unlocked;
1st	Not given, La Rochelle, 1976 [11];	Free of charge;	Low security;
(1965–1994)	Not given, Cambridge, 1993 [11];	Distinct.	No riding guideline.
	Farsϕ and Gren$\dot{a}$, Denmark, 1991 [20].		
	Bycyklen, Copenhagen, 1995 [11];	Locked;	Non real-name verification;
2nd	V$\overset{´}{e}$lo $\overset{`}{a}$ la Carte, Rennes, 1998 [19];	Specific stations;	No location information;
(1995–2005)	Call a Bike, Munich, 2001 [21];	Payment system;	High production cost.
	V$\overset{´}{e}$lo‘v, Lyon, 2004 [22].	Coin or Smart card access.	
	V$\overset{´}{e}$lib‘, Paris, 2007 [23];	Record riding time;	Low accuracy;
3rd	UseBike, San Paulo, 2008 [24];	GPS track;	Limited battery life;
(2006–2013)	Smart Bike, Washington DC, 2008; [25];	Networking;	Fixed lock password.
	HZBike, Hangzhou, 2008 [26].	Information service.	
	OFO, Beijing, 2014 [27];	Smart Lock;	Difficult maintenance;
4th	Mobike, Shanghai, 2015 [28];	APP on smart phone;	Environment constrains;
(2014-)	Hellobike, Xiamen, 2016 [29];	Rechargeable battery;	Unreasonable allocation.
	oBike, Singapore, 2017 [30].	Cellular network.	

**Table 3:** Examples of present shared bicycle in China (accessed by July 2017).

Brands	HZBike (in Hangzhou)	OFO	Mobike
**Pictures**	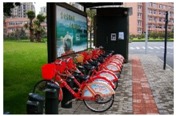	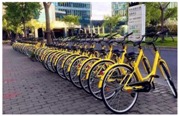	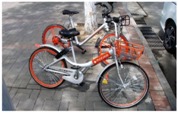
**Types**	With Dock	Without Dock	Without Dock
**Operators**	Government	OFO Inc.	Mobike Inc.
**Bicycles**	>858,000	>7,500,000	>1,000,000
**Total Rent Times**	>700,000,000	>500,000,000	>400,000,000
**Dock Stations**	>3000		

**Table 4:** Price comparison among three brands.

Brands	HZBike	OFO	Mobike
**Deposit**	199 CNY	199 CNY	299 CNY
**Rent**	Free in first hour;	1 CNY per hour	1 CNY per hour
	1 CNY per extra one hour		

**Table 5:** Different designs among three brands [41,42].

Brands	HZBike	OFO	Mobike
**Weight**	<20 kg	<20 kg	>20 kg
**Pneumatic tire**	Yes	No	No
**Adjustable seat**	No	Yes	Yes

**Table 6:** Comparison on cost and maintenance of three brands [41,42].

Brands	HZBike	OFO	Mobike
**Maintenance**	Fine	Bad	Bad
**Materials**	Common iron	Common iron	Aluminum alloy
**Cost**	About 450 CNY	About 600 CNY	About 1000 CNY

**Table 7:** Comparison of security on three brands [26,27,43].

Brands	HZBike	OFO	Mobike
**Brake**	Caliper brakes + Band brake	Band brake	Disc brake
**Payment**	RFID	QR-code	QR-code
**Operator**	Government	Enterprise	Enterprise
